# On Hereditary Transmission of Phthisis

**Published:** 1852-11

**Authors:** M. Guillot


From L' Union Medicale.

*On Hereditary transmission of Phthisis, by* M. GUILLOT.

SINCE 1825, M. Guillot has been tracing out the history of certain cases of phthisis, in order to illustrate the laws which regulate the hereditary transmission of this disease. He follows the history of the family line, in order to ascertain whether this does not, by successive degradation, become exhausted and extinguished. He refers to the case of a man who died of phthisis, aged 66. Before the age of 48 all his four children died of the same disease; all had children, but the third generation did not survive the period of the first dentition, all being carried off either by pneumonia supervening on tubercle, or by tubercular meningitis. In another example, a grandfather died of phthisis. One of his daughters also died of it at 30. The other daughter is still living, but three of her children have died either of tubercular pneumonia, or meningitis. The general conclusion is, that in proportion as phthisis descends in the genealogical scale, its manifestation takes place at an earlier period in life. A child will run greater chance, therefore, of falling a victim to the consequences of the numerous accidents of a tubercular affection, in proportion as the phthisical parents who have given birth to it, have not attained advanced age. In a diagnostical point of view, then, the existence of tubercular disease in the offspring, while yet young, offers a very strong presumption of phthisis. The practical importance of this is especially evident in pneumonia, so common is it to find tubercles of the lungs in the bronchial glands, masked by the signs of this affection.

